# Clinical and Radiologic Considerations for Idiopathic Superior Mesenteric Artery Syndrome

**DOI:** 10.7759/cureus.1822

**Published:** 2017-11-05

**Authors:** Mina S Makary, Anand Rajan, Anthony M Aquino, Suresh K Chamarthi

**Affiliations:** 1 Radiology, The Ohio State University Wexner Medical Center, Columbus, Ohio, Usa; 2 College of Medicine, The Ohio State University Wexner Medical Center, Columbus, Ohio, Usa

**Keywords:** sma syndrome, superior mesenteric artery, duodenal obstruction, wilkie’s syndrome

## Abstract

Superior mesenteric artery (SMA) syndrome often occurs in the setting of rapid weight loss and scoliosis corrective spinal surgery. A reduction of fat around the third part of the duodenum can predispose the duodenum to compression and obstruction by the SMA as it emerges from the abdominal aorta. In this report, we describe this underdiagnosed condition in a previously healthy young female presenting with progressive post-prandial emesis, non-specific abdominal pain, and weight loss. A critical review of this disease process is explored to highlight pathology, imaging characteristics, and essential alternative diagnostic considerations. We also discuss potential complications and current treatment strategies. SMA syndrome poses unique diagnostic challenges, and an awareness of its clinical presentation can further improve patient outcomes and avoid potentially life-threatening complications.

## Introduction

Superior mesenteric artery (SMA) syndrome, or vascular compression of the duodenum, presents as a constellation of gastrointestinal symptoms that resembles small bowel obstruction. It is typically described in the setting of rapid weight loss, wasting conditions, such as trauma and burns, and corrective spinal surgery. The prevalence of this underdiagnosed condition is estimated to be 0.3% to 2.4% with a higher prevalence in women [1]. Due to the rarity of this syndrome and the difficulty of diagnosis, there is a paucity of reports in the literature describing varying presentations as well as medical and surgical treatment options. Previous reports by Suhani, et al. and Derrick, et al. described patients with rare anatomic causes, including short and hypertrophic ligament of Treitz who responded to surgical treatment, while Altiok, et al. presented cases of SMA syndrome secondary to spinal deformities [2-4]. Recent studies by Sun, et al. demonstrated successful, minimally invasive duodenojejunostomy techniques for treatment in select patients [5]. In this case study, we report a patient with an insidious onset of SMA syndrome without known risk factors who responded to primary medical management.

## Case presentation

A 27-year-old healthy female presented with a three-month history of worsening postprandial nausea, emesis, and weight loss. She experienced early satiety with meals, and her symptoms would last from several hours to days. Her bowel movements were also greatly impaired compared to her baseline. Her physical exam was unremarkable, and the laboratory workup, including basic metabolic panel, hematologic panel, and microbiologic stool studies, were within normal limits. Additional workup, including esophagogastroduodenoscopy (EGD), video capsule endoscopy, and gastric emptying studies, were also unremarkable.

**Figure 1:**
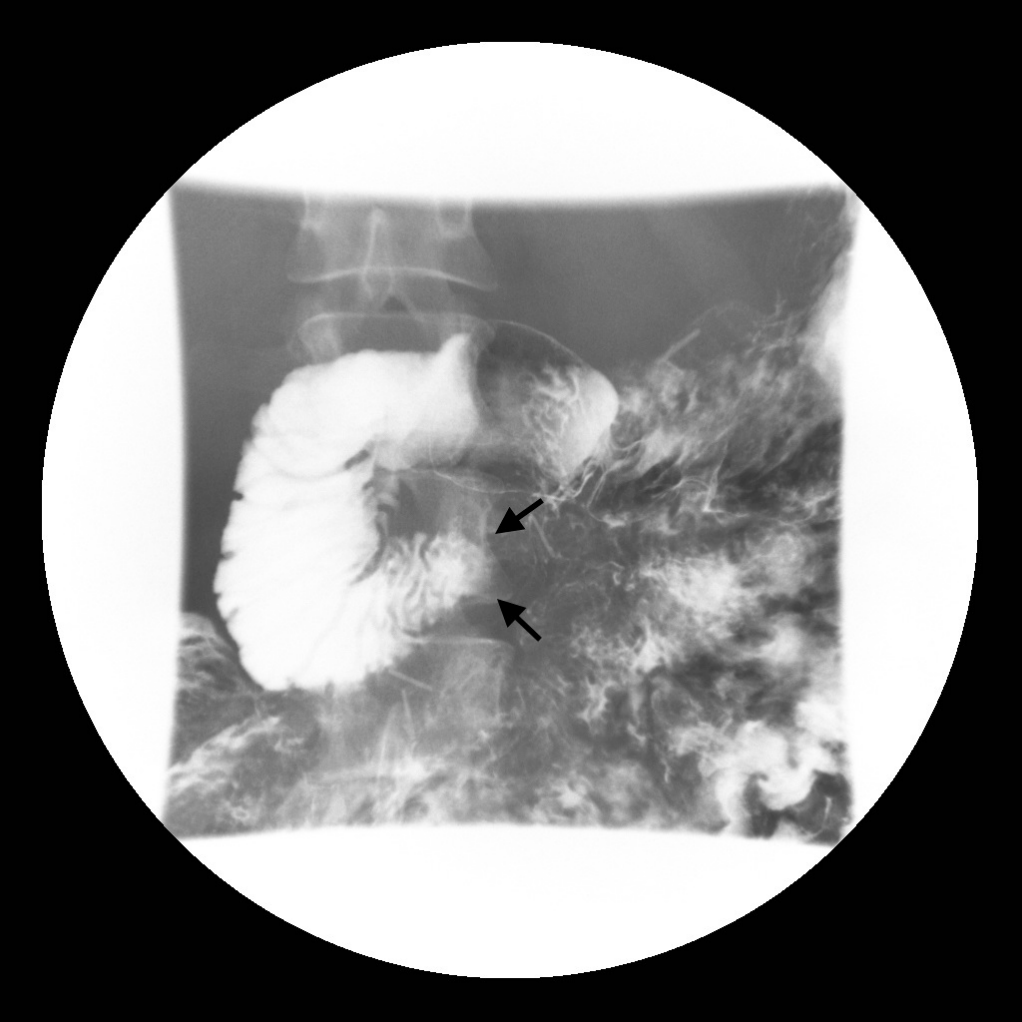
Fluoroscopic image of the abdomen following barium intake demonstrates contrast pooling in the first and second parts of the duodenum with an abrupt cut-off at its third part (arrows), coinciding with the superior mesenteric artery impression.

**Figure 2:**
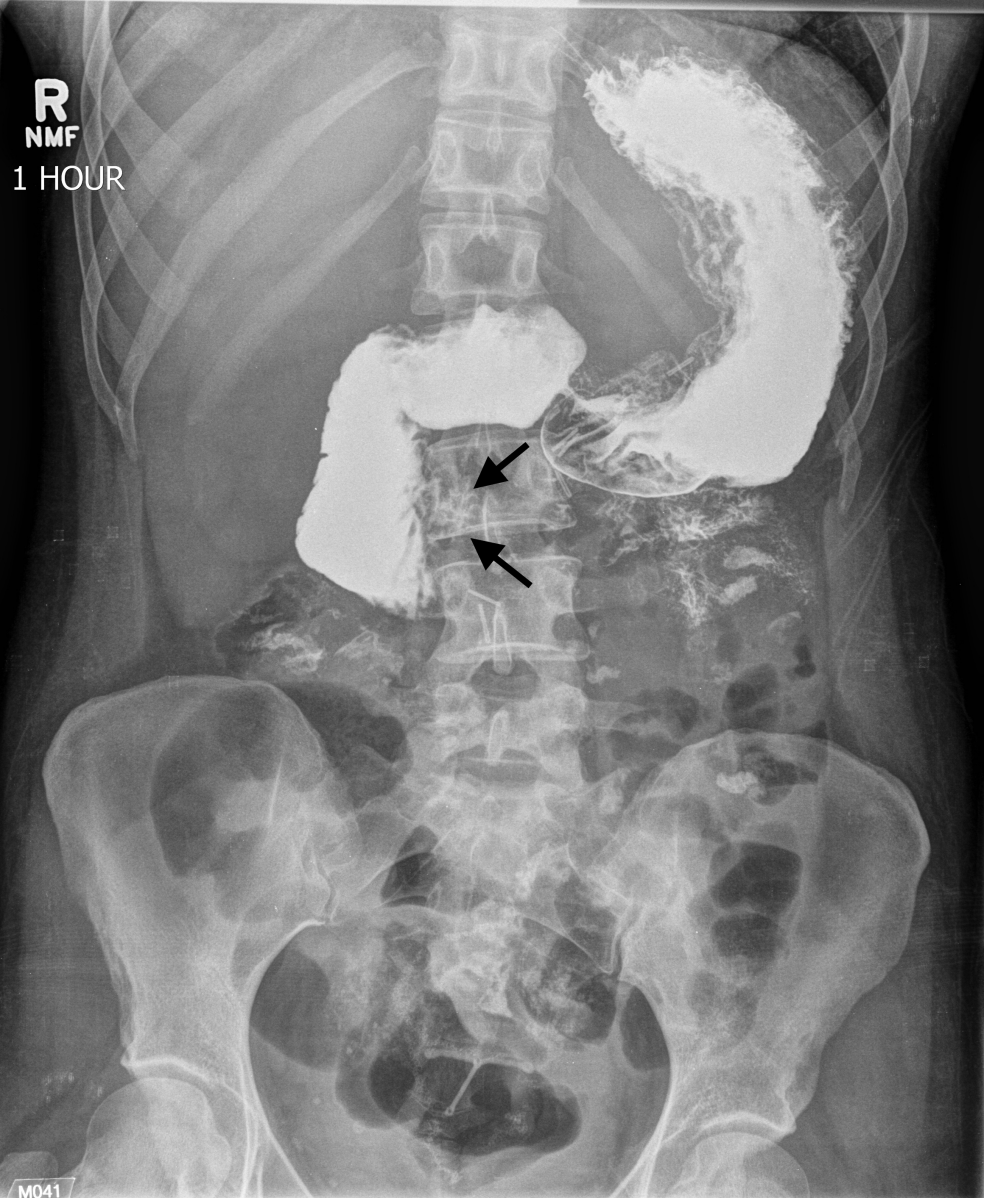
Abdominal radiograph after one-hour demonstrate persistent contrast pooling in the proximal duodenum with an abrupt cut-off at its third part (arrows), coinciding with the superior mesenteric artery impression.

**Figure 3:**
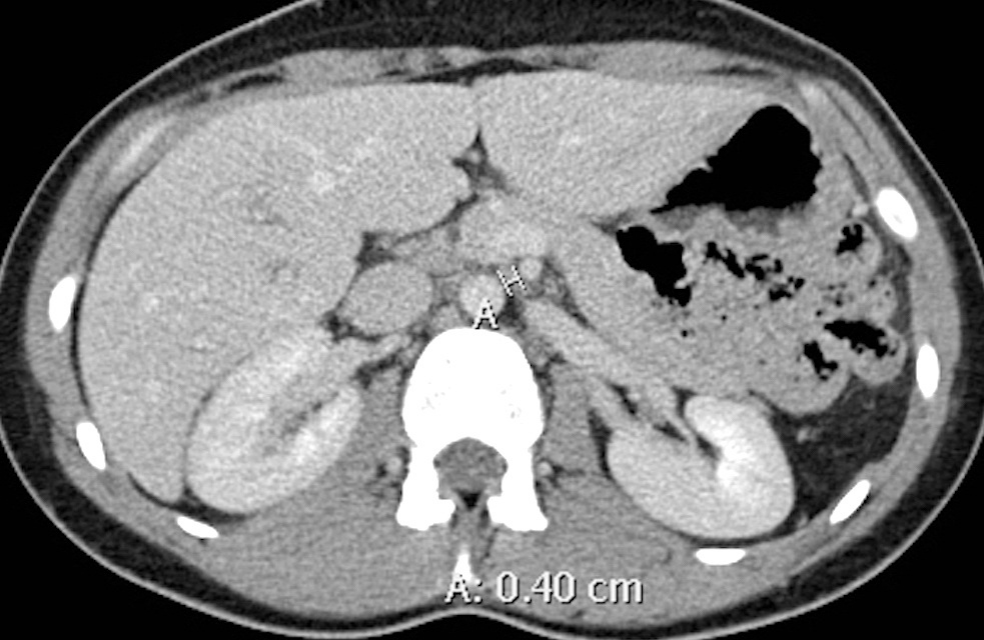
Contrast-enhanced axial CT image demonstrates a narrow aortomesenteric distance of 4 mm (calipers) (normal > 8 mm).

**Figure 4:**
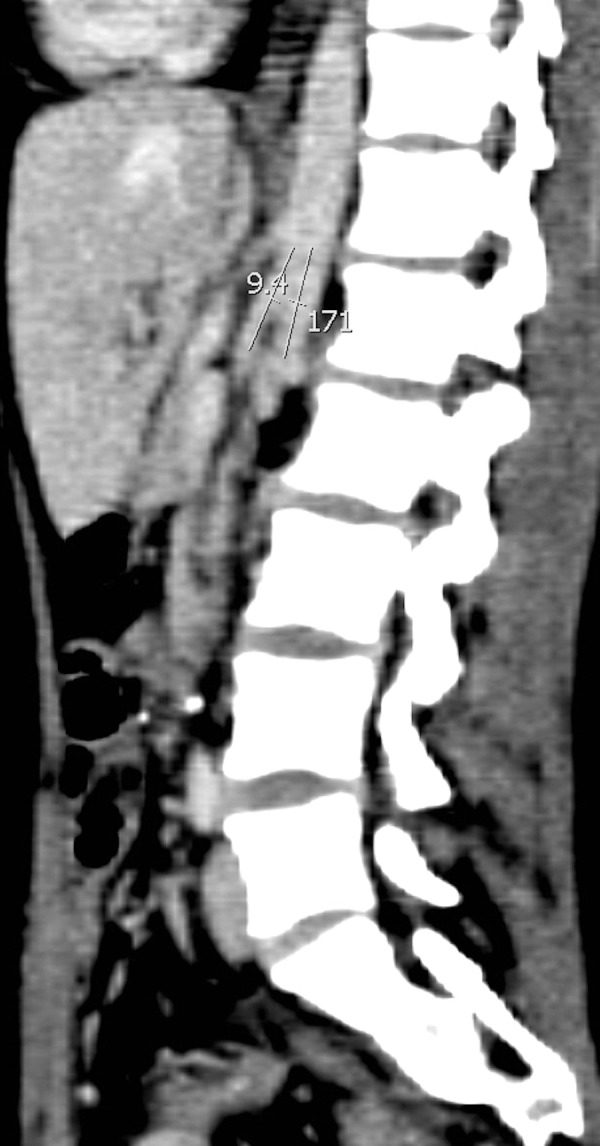
Contrast-enhanced sagittal CT image demonstrates a narrow aortomesenteric angle of 9° (calipers) (normal > 22°).

A fluoroscopic small bowel follow-through study was obtained to assess her bowel motility and demonstrated contrast accumulation into a dilated second part of the duodenum while the third and fourth parts were initially poorly distended with contrast (Figures 1-2). With prone positioning, increased passage of contrast was noted into the second and third parts of the duodenum. The findings of both studies were consistent with SMA syndrome and were confirmed with an abdominal computed tomography (CT) scan demonstrating narrowed aortomesenteric distance and angle (Figures 3-4).

Multidisciplinary evaluation by our internal medicine, gastroenterology, and general surgery teams evaluated the patient and recommended a trial of conservative treatment before considering surgical options. The patient was treated with appetite stimulations, antiemetics, and promotility agents which improved her symptoms. Her weight returned to baseline and she has remained asymptomatic to date.

## Discussion

SMA syndrome is a rare but serious condition that classically presents with nonspecific gastrointestinal symptoms and can lead to severe complications if not recognized or misdiagnosed. The SMA originates from the descending aorta at the L1 spinal cord level and descends inferiorly, forming an acute angle with the descending aorta, referred to as the aortomesenteric angle. The SMA normally emerges from the aorta at an angle of 38-65 degrees [2]. SMA syndrome occurs when the aortomesenteric angle decreases, resulting in vascular compression of the duodenum and presenting symptoms of small bowel obstruction. Patients often complain of postprandial epigastric pain, early satiety, bilious emesis, significant weight loss, nausea, and gastric reflux. Some patients report alleviation of their symptoms with positional variation, specifically in the prone position [5].

While SMA syndrome can occur due to congenital or acquired risk factors, an estimated 40% of cases occur idiopathically, such as in this case [1]. A congenitally short ligament of Treitz, which suspends the distal duodenum, can result in congenital SMA syndrome [5]. Depletion of the retroperitoneal fat surrounding the duodenum causes a decreased aortomesenteric distance, thus predisposing patients with rapid weight loss (bariatric surgery, trauma, burns, malignancy, malabsorption, and eating disorders) to SMA syndrome [4]. Additional common causes include aortic aneurysm repair and corrective spinal surgery for scoliosis with an estimated prevalence in this patient population up to 2.4% [6]. Other reported risk factors include acquired immune deficiency syndrome (AIDS) and malignancy [7]. In this report, we describe a patient with idiopathic presentation and no known risk factors. This cohort of patients represent the most challenging group of patients to diagnose, given the non-specific symptoms, but are critical to diagnose early, given the positive response to treatment.

The diagnosis of SMA syndrome is one of exclusion, made with high clinical suspicion followed by confirmatory imaging. Since most patients present with nonspecific abdominal pain and obstructive symptoms, the differential diagnosis is broad and includes small bowel obstruction, gastroparesis, pancreatitis, peptic ulcer disease, and mesenteric ischemia. The imaging workup starts with radiography as the first modality of choice but findings can vary from normal to an obstructive bowel gas pattern. The key in identifying SMA syndrome is obtaining a small bowel follow-through study to fluoroscopically visualize the bowel behavior in real-time, particularly demonstrating improved contrast passage in the prone position compared to the supine state. This was the key test, helping clinch the diagnosis for our patient, who did not have the classic risk factors or presentation history for SMA syndrome. In patients with equivocal fluoroscopic findings, Haye’s maneuver, in which the flow of contrast into the jejunum improves when the patient is in the left lateral position, can also be used to increase the specificity of the study [1, 7-8].

In addition to excluding other conditions in the differential diagnosis, a contrast-enhanced abdominal CT is further used to evaluate the anatomy and confirm the diagnosis of SMA syndrome. The aortomesenteric angle and distance can be directly measured on a sagittal CT reconstruction. Values of < 8 mm for the aortomesenteric distance and < 22° for the aortomesenteric angle are reported to correlate strongly with clinical symptoms [9]. Both of these criteria were met in the CT imaging obtained for the patient in this report and helped confirm the diagnosis suggested by fluoroscopic imaging. Additional benefits of cross-sectional imaging include visualizing the extent of duodenal compression, quantifying the amount of retroperitoneal fat, identifying potential complications, and (if necessary) evaluating anatomy for surgical planning.

The treatment for SMA syndrome is initially non-surgical, with surgical options available for recalcitrant symptoms. The patient in this report responded to primary medical management without surgical intervention. Primary conservative management includes fluid replacement, enteral/parenteral feeding, appetite stimulations, antiemetics, and promotility agents, as needed, with the ultimate goal of weight gain to allow for retroperitoneal fat pad replenishment [3]. In the acute phase, nasogastric (NG) decompression and postural changes after eating are recommended to reduce the degree of obstruction and facilitate the passage of duodenal contents [3]. When medical management fails, surgical options, such as duodenojejunostomy, gastroduodenostomy, and Strong’s procedure (lysis of the ligament of Treitz to free the duodenum from the aortomesenteric space), are considered [5]. Duodenojejunostomy is the preferred surgical approach due to lower rates of surgical complications, including ulcerations and failure to relieve the obstruction [5]. The procedure is performed by creating a duodenojejunostomy anastomosis around the duodenum obstructed by the SMA to bypass the obstructed segment.

Despite the excellent treatment outcomes, adverse events, unfortunately, do occur. Severe dehydration and life-threatening electrolyte imbalance are the most common complications in patients with delayed treatment. Late recognition and management can also lead to chronic aspiration and, potentially, acute respiratory distress syndrome (ARDS) due to progressive gastric content reflux. Peptic ulcers and bowel perforation, leading to fatal consequences, have been reported in rare cases [1]. Finally, some patients with severe cases of SMA syndrome may have concurrent compression of the left renal vein between the aorta and SMA (nutcracker syndrome), which may lead to left flank pain and hematuria [10].

## Conclusions

SMA syndrome is a rare disease that causes proximal small bowel obstruction due to the narrowing of the SMA angle with the aorta compressing the third part of the duodenum. This entity is frequently idiopathic, but predisposing factors include weight loss, spine surgery, and open aortic aneurysm repair. It is often misdiagnosed or suboptimally treated due to the non-specific presenting symptoms. This report described a previously healthy young female presenting with progressive post-prandial emesis, non-specific abdominal pain, and weight loss. She is one of the 40% of SMA syndrome patients who presented with an insidious onset without known risk factors. Early recognition and obtaining appropriate cross-sectional and fluoroscopic imaging is key to achieving a good prognosis and avoiding potentially life-threatening complications. The patient in this report responded to primary medical management, which is the initial treatment approach, including gastric decompression and conservative therapy. Surgical intervention could be required for patients with persistent symptoms.
